# Proton-Blocking Anion-Exchange Membranes for Efficient Lithium Hydroxide Recovery by Bipolar Membrane Electrodialysis

**DOI:** 10.3390/membranes16010008

**Published:** 2025-12-30

**Authors:** Ji-Hyeon Lee, Moon-Sung Kang

**Affiliations:** Department of Green Chemical Engineering, College of Engineering, Sangmyung University, Cheonan 31066, Republic of Korea; 2021d3009@sangmyung.kr

**Keywords:** bipolar membrane electrodialysis, anion-exchange membrane, proton-blocking, brominated poly(phenylene oxide), diamines, lithium hydroxide recovery

## Abstract

In bipolar membrane electrodialysis (BPED), proton transport through the anion-exchange membrane (AEM) is a major factor that reduces overall process efficiency. In this study, we propose a composite AEM incorporating a proton-blocking layer that combines strongly basic and weakly basic functional groups on top of a strongly basic AEM, providing proton-blocking capability while minimizing degradation of membrane conductivity. The proton-blocking layer is prepared by reacting brominated poly(phenylene oxide) (BPPO) with diamines having different alkyl chain lengths, namely *N*,*N*,*N′*,*N′*-tetramethyl-1,6-hexanediamine (TMHDA), *N*,*N*,*N′*,*N′*-tetramethyl-1,3-propanediamine (TMPDA), and *N*,*N*,*N′*,*N′*-tetramethylethylenediamine (TMEDA). When TMHDA, which has the longest alkyl chain, is introduced into PPO, the resulting membrane exhibits high conductivity but low proton-blocking performance. In contrast, when TMEDA, which has the shortest alkyl chain, is introduced, the membrane shows low conductivity and high proton-blocking performance. Therefore, the balance between membrane conductivity and proton-blocking performance can be optimized by adjusting the molar ratio of the two diamines. The composite AEM prepared with the optimal composition simultaneously demonstrates superior conductivity and proton-blocking performance compared to the commercial proton-blocking membrane (ACM, Astom Corp., Tokyo, Japan). Furthermore, the application of this membrane has been shown to effectively improve both the energy efficiency and current efficiency of the BPED process for lithium hydroxide recovery.

## 1. Introduction

Since the 1990s, lithium-ion batteries (LIBs) have been widely used in various mobile electronic devices, such as smartphones and laptops. Recently, the demand for LIBs in electric vehicle (EV) applications has increased rapidly, resulting in a significant rise in the production of key materials for LIB manufacturing, including cathode materials [1,2]. Meanwhile, lithium hydroxide (LiOH) is one of the fundamental raw materials used in the production of cathode materials. Its ability to readily react with nickel makes it suitable for synthesizing high-energy-density cathode materials. Consequently, LiOH is primarily used in the manufacture of high-density, high-capacity EV batteries. With the rapid expansion of the LIB industry, the demand for LiOH has also been increasing rapidly [3]. However, conventional solvent extraction and chemical precipitation processes for LiOH production require large amounts of chemical reagents and generate sludge, leading to significant environmental concerns. Therefore, the development of more environmentally friendly and efficient technologies for lithium separation and LiOH production is essential [4].

Representative methods for separating trace metal ions include membrane processes such as nanofiltration (NF) and reverse osmosis (RO), as well as electro-membrane processes such as electrodialysis (ED), electrodialysis reversal (EDR), and bipolar membrane electrodialysis (BPED). Among these, BPED has attracted attention as an environmentally friendly and efficient technology capable of recovering lithium ions with high purity from seawater or brine solutions [5,6]. BPED systems have been widely used to generate acids and bases from salt solutions because they can efficiently convert salts into their corresponding acids and bases without the addition of external reagents [7]. A typical BPED stack consists of a cation-exchange membrane (CEM), an anion-exchange membrane (AEM), and a bipolar membrane (BPM). When an electric potential is applied, water splitting occurs at the bipolar junction of the BPM, producing H^+^ and OH^−^ ions, which then participate in the formation of acids and bases, respectively. For example, from a solution containing Li^+^ ions, LiOH and an acid (HCl or H_2_SO_4_) can be effectively produced through the BPED process [8]. At this stage, the AEM blocks the permeation of H^+^ ions generated in the BPM, thereby preventing excessive proton transport into the base compartment. However, because protons have an extremely small ionic radius (0.282 nm) and high mobility (350 Ω^−1^ cm^−2^ mol^−1^), they can easily pass through the AEM. As a result, some protons can also penetrate the CEM and hinder the formation of LiOH in the base compartment. This undesirable proton transport through the AEM, which is commonly referred to as “proton leakage”, reduces the concentrations of the recovered acid and base and decreases the current efficiency of the BPED process [9,10,11]. Therefore, developing an AEM with a high proton-blocking capability is essential for improving the overall performance of BPED.

Proton transport in ion-exchange membranes (IEMs) is generally explained by two mechanisms: the vehicle mechanism and the Grotthuss mechanism. In the vehicle mechanism, protons bind to water molecules to form hydration clusters and then move by diffusion. Meanwhile, in the Grotthuss mechanism, protons are transferred to adjacent water molecules through the continuous breaking and reforming of hydrogen bonds. In both mechanisms, water molecules act as essential mediators of proton transport [12,13,14,15,16]. Proton leakage mainly results from the proton adsorption capacity and high proton mobility of strongly basic functional groups, and this leakage can be mitigated by reducing the membrane water content. To achieve this, several research strategies have been reported, including the introduction of hydrophobic functional groups, an increase in the degree of cross-linking, and the substitution of strongly basic quaternary ammonium groups with weakly basic anion-exchange groups [11,12,13,15,16,17,18]. In particular, AEMs containing weakly basic amine groups exhibit weaker hydration effects than membranes with strongly basic quaternary ammonium groups, leading to lower swelling and reduced water uptake (WU) [13,15,17]. It has also been reported that protonated weakly basic groups impart proton-blocking ability to AEMs through electrostatic repulsion with protons [17]. In addition, introducing weakly basic functional groups into side chains grafted onto the polymer backbone can induce the formation of microphase-separated structures due to polarity differences between the hydrophilic and hydrophobic segments. This microphase separation creates nanoscale ion-conducting channels that facilitate the transport of counterions, thereby improving ion conductivity while enhancing the Donnan exclusion effect against protons [18].

The proton-blocking performance of an AEM can generally be improved by reducing its WU. However, this reduction in WU often causes a decrease in ion conductivity. Therefore, it is necessary to develop a design strategy for proton-blocking AEMs that can overcome the trade-off between ion conductivity and proton-blocking capability [19]. For instance, increasing the ion-exchange capacity (IEC) of an AEM can enhance Donnan exclusion, but a higher IEC also tends to increase WU and proton leakage. Previous studies have attempted to improve the proton-blocking properties of AEMs by introducing various weakly basic functional groups. Examples include 2-(*N*,*N*-dimethylamino)ethyl methacrylate (DMAEMDA) [11,12], dipentylamine [13], butyldimethylamine [18], 1,4-diazabicyclo [2.2.2]octane (DABCO) [20], *N*,*N*-dimethylallylamine (DMAA) [15], 1,3-di-4-piperidylpropane (DiPRD), *N*-methylpiperidine (MPRD) [19], 4-acetylpyridine [20], crosslinked (3-acrylamidopropyl)trimethylammonium chloride and 1,4-bis(acryloyl)piperazine (AAP–BAP) [21], 1-methyl-4-piperidone [22], and *N*-butylimidazole (*N*–BNI) [23]. The introduction of these weakly basic functional groups, together with the polarity differences between the hydrophilic and hydrophobic segments, induces microphase separation and self-crosslinking. This structural effect suppresses membrane swelling while simultaneously providing a high IEC and excellent proton-blocking capability.

Based on these previous research findings, this study proposes a composite AEM that incorporates a thin proton-blocking layer on the surface of an AEM containing strongly basic functional groups to more effectively mitigate the trade-off between ion conductivity and proton-blocking capability. This approach functionalizes only the membrane surface, thereby achieving both a high ion flux and enhanced proton-blocking performance. The proton-blocking layer proposed in this study is based on a poly(2,6-dimethyl-1,4-phenylene oxide) (PPO) backbone. Side chains containing diamines with different alkyl chain lengths, namely, *N*,*N*,*N′*,*N′*-tetramethyl-1,6-hexanediamine (TMHDA, C6), *N*,*N*,*N′*,*N′*-tetramethyl-1,3-propanediamine (TMPDA, C3), and *N*,*N*,*N′*,*N′*-tetramethylethylenediamine (TMEDA, C2), were introduced, and their respective performances were compared. Furthermore, the ion conductivity and proton-blocking performance of the membrane were optimized by adjusting the composition ratio of the diamines. The fabricated composite AEM contains both strongly basic and weakly basic functional groups, and the introduction of an alkyl spacer ensures high ion conductivity while maintaining a low WU. In particular, the thin proton-blocking layer formed on the base membrane, which was prepared by filling an ionomer into a porous polyethylene (PE) support used as a battery separator, enhanced the mechanical stability of the membrane while also providing proton-blocking properties. As a result, the composite AEM developed in this study exhibited superior mechanical strength, high proton-blocking capability, and improved ion conductivity compared with a commercial proton-blocking membrane, indicating its potential to enhance lithium recovery efficiency in the BPED process.

## 2. Materials and Methods

### 2.1. Materials

Vinylbenzyl trimethylammonium chloride (VTAC) and styrene (Sty) were used as monomers to prepare the anion-exchange polymer for pore filling. Trimethylolpropane triacrylate (TMPTA) served as a cross-linker, and diphenyl(2,4,6-trimethylbenzoyl)phosphine oxide (TPO) was used as a photoinitiator. PPO, bromine, and chlorobenzene were employed to prepare brominated PPO (BPPO). TMHDA, TMPDA, and TMEDA were used to introduce diamine functional groups with different alkyl chain lengths into the membrane. All reagents were obtained from Sigma-Aldrich Corp. (St. Louis, MO, USA) and used without further purification.

A 25 μm-thick PE separator (Hipore, Asahi Kasei E-Materials Corp., Tokyo, Japan) was used as the support for the pore-filled membranes. For comparison of proton-blocking performance, ACM (Astom Corp., Tokyo, Japan) was selected as a commercial proton-blocking AEM. In addition, CM-2 (Astom Corp., Tokyo, Japan), a commercial CEM, and BPU (Astom Corp., Tokyo, Japan), a commercial BPM, were used in the BPED experiments.

### 2.2. Preparation of Surface Modified Membranes

#### 2.2.1. Preparation of Pore-Filled AEM

The pore-filled AEM (PFAEM), used as the base membrane, was fabricated using a porous PE substrate through monomer filling and photopolymerization. The molar ratio of the monomers, VTAC and Sty, was fixed at 1:1. The crosslinker (TMPTA) and the photoinitiator (TPO) were added at 10 wt% and 5 wt%, respectively, based on the total monomer weight. Ethanol was used as a cosolvent to dissolve all components. The monomer mixture was impregnated into the porous PE support, which was then laminated between two release films. Photopolymerization was carried out for 17 min using a 1 kW UV irradiator (UV-CB-1.5X1, Wonil Science, Daejeon, Republic of Korea) [24].

#### 2.2.2. Bromination of PPO

For the bromination of PPO, a 500 mL four-necked flask equipped with a condenser, nitrogen inlet, thermometer, and dropping funnel connected to a chiller maintained at −10 °C was used. PPO (16.0 g, 8 wt%) was dissolved in chlorobenzene (184 g) under a nitrogen atmosphere, and the reaction temperature was kept at 131 °C. A solution of bromine (21.3 g) diluted in chlorobenzene (85.2 g) was added dropwise through the funnel with stirring. After the addition, the mixture was reacted at 131 °C for 10 h. The resulting BPPO solution was then added dropwise to methanol to precipitate the product, which was washed several times to remove unreacted residues and dried at 80 °C for 24 h to obtain BPPO [25]. The structural characteristics of the prepared BPPO were examined through nuclear magnetic resonance (NMR, AVANCE III 400, Bruker, Billerica, MA, USA) analysis. The ^1^H NMR spectrum of the prepared BPPO is shown in Figure S1. The peak integration values were applied to Equation (1) to calculate the degree of bromination (DB) [26,27,28]:

(1) $$DB(\%)=\frac{{3I}_{\left(-{CH}_{2}Br\right)}}{{2I}_{\left(-{CH}_{3}\right)}+{3I}_{\left(-{CH}_{2}Br\right)}}\times 100$$

where $I_{\left(-{CH}_{2}Br\right)}$ is the integration value of the proton peak corresponding to benzylic bromine, and $I_{\left(-{CH}_{3}\right)}$ is the integration value of the methyl proton peak. As a result, the DB of the prepared BPPO was determined to be approximately 55%.

#### 2.2.3. Reaction of BPPO and Diamines

First, BPPO (1 g, 0.005 mol) was dissolved in *N*-methyl-2-pyrrolidone (NMP, 15 mL) to prepare a BPPO solution. A diamine solution was prepared by dissolving 0.0005 mol each of TMEDA, TMPDA, and TMHDA in NMP (20 mL). The BPPO solution was then slowly added to the diamine solution and stirred at room temperature for 6 h. After the reaction was complete, the mixture was precipitated in isopropyl ether, washed at least three times, and vacuum-dried at room temperature to obtain the PPO–diamine polymer. A series of polymers were prepared using the same method, keeping the total diamine amount constant at 0.0005 mol while adjusting the molar ratios of TMHDA and TMEDA to 10:0, 9:1, 7:3, 5:5, and 0:10.

#### 2.2.4. Fabrication of Composite Membranes

**Figure 1:**
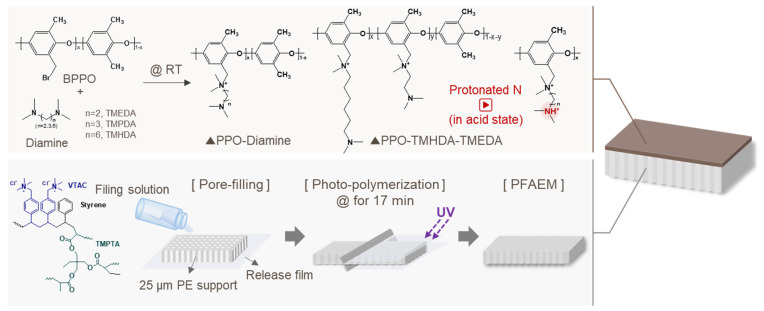
Schematic illustration of the preparation of PPO-diamine polymers and fabrication process of the composite AEMs.

The diamine-modified PPO was dissolved in dimethylacetamide (DMAc) at a concentration of 20 wt% to prepare a coating solution. The base membrane (PFAEM) was fixed on a glass substrate, and the coating solution was cast onto its surface using a bar coater (Knife Coating Device KP-3000V, KIPAE, Suwon, Republic of Korea). The coated membrane was then dried in an oven at 60 °C to obtain the composite membrane. The preparation of PPO-diamine polymers and fabrication process of the composite AEMs are illustrated in Figure 1. The deprotonated and protonated membranes were prepared using the composite membranes fabricated under the same conditions described above. The deprotonated membrane was obtained by immersing the composite membrane in a 0.5 M NaCl aqueous solution at room temperature for 24 h, followed by thorough washing with distilled water to remove any residual salts. For the protonated membrane, the composite membrane was immersed in a 0.34 M H_2_SO_4_ (or 0.5 M HCl) solution at room temperature for 24 h to protonate the tertiary amine groups. Both membranes were stored in distilled water before use.

### 2.3. Membrane Characterizations

The surface and cross-sectional morphologies of the membranes were examined using field emission scanning electron microscopy (FE-SEM, SIGMA 500, Carl Zeiss, Jena, Germany). Fourier transform infrared spectroscopy (FT-IR, FT/IR-4700, Jasco, Tokyo, Japan) was used to analyze the chemical structures. The *d*-spacing and chemical bonding states of the modified polymers were characterized using X-ray diffraction (XRD, D2 Phaser, Bruker, Billerica, MA, USA) and X-ray photoelectron spectroscopy (XPS, K-Alpha, Thermo Fisher Scientific, Waltham, MA, USA). The surface hydrophilicity of the membranes was evaluated using a contact angle analyzer (Phoenix 150, SEO Co., Suwon, Republic of Korea). The electrical resistance (ER) of the membranes was measured in 0.5 M NaCl, HCl, H_2_SO_4_, and Li_2_SO_4_ aqueous solutions using a lab-made two-point probe clip cell connected to an impedance analyzer (SP-150, Bio-Logic Science Instruments, Seyssinet-Pariset, France). The ER values were calculated using Equation (2) [29]:

(2) $$\mathrm{ER}\;(\Omega \cdot {cm}^{2})={(\left|Z\right|}_{sample}\cdot \cos\theta_{sample}-{\left|Z\right|}_{blank}\cdot \cos\theta_{blank})\times A$$

where *|Z|* is the impedance magnitude (Ω), *θ* is the phase angle, and *A* is the effective membrane area (0.196 cm^2^). The transport number (*t^−^*), which represents the selectivity of ion transport, was measured by the electromotive force (*emf*) method using a two-compartment diffusion cell and calculated using Equation (3) [30,31]:

(3) $$E_{m}(-)=\frac{RT}{F}(1-2t^{-})\ln\frac{C_{L}}{C_{H}}$$

where *E_m_* is the measured cell potential, *R* is the gas constant, *T* is the absolute temperature, *F* is the Faraday constant, and *C_L_* and *C_H_* are the concentrations of the NaCl solutions, which were 1 mM and 5 mM, respectively. The cell potential was measured using a pair of Ag/AgCl electrodes connected to a digital voltmeter. Chronopotentiometry experiments were conducted to assess the membrane’s stability in highly acidic solutions. For this purpose, a two-compartment cell identical to that used for the transport number measurements was utilized. To measure the membrane potential, a pair of Ag/AgCl reference electrodes was positioned close to the membrane surface, and a pair of Pt plate electrodes was used to supply a constant current. Each chamber was filled with 150 mL of 1 M H_2_SO_4_ solution, and the membrane potential change over time was recorded while a constant current density of 30 mA cm^−2^ was applied using a potentiostat/galvanostat (SP-150, Bio-Logic Science Instruments, Seyssinet-Pariset, France) at 40 °C. The WU was calculated by substituting the measured wet weight (*W_wet_*) and dry weight (*W_dry_*) of the membrane into Equation (4) [32]:

(4) $$WU(\%)=\;\frac{W_{wet}-W_{dry}}{W_{dry}}\;\times 100\;$$

The IEC was determined using the conventional Mohr titration method. The membrane was first immersed in a 0.5 M NaCl solution to reach equilibrium, rinsed with distilled water, and then immersed in a 0.25 M Na_2_SO_4_ solution to replace the Cl^−^ ions within the membrane with SO_4_^2−^ ions. The amount of displaced Cl^−^ in the solution was titrated with a 0.01 M AgNO_3_ standard solution using K_2_CrO_4_ as an indicator. The measured Cl^−^ concentration was then substituted into Equation (5) to calculate the IEC [29].

(5) $$IEC\left(\frac{meq}{g}\right)=\frac{C_{{Cl}^{-},sample}\;\cdot \;V_{sample}}{W_{dry}}$$

### 2.4. Proton-Blocking Performance Evaluation Test

**Figure 2:**
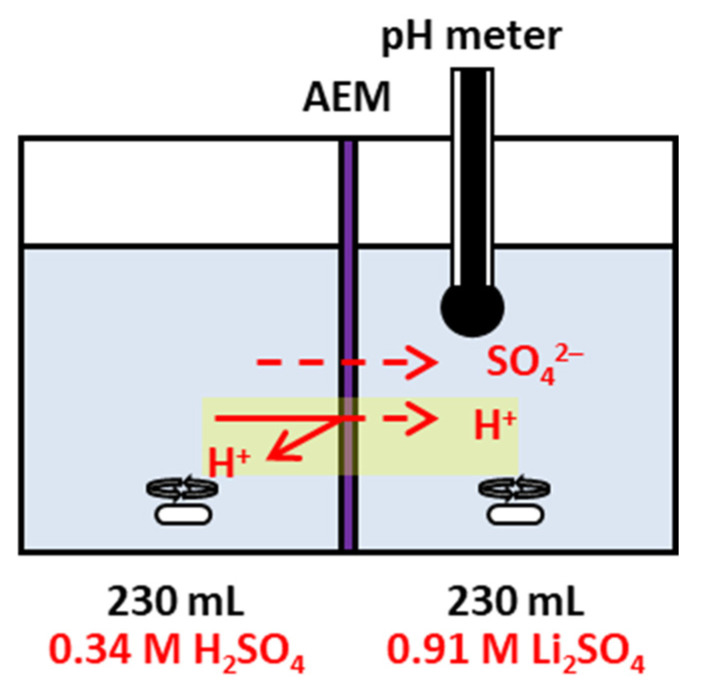
Schematic diagram of the proton-blocking test setup and measurement configuration.

The proton-blocking performance of the fabricated membranes was evaluated using a lab-made two-compartment diffusion cell, as shown in Figure 2. The membrane under test was placed between the two compartments and securely clamped. A 0.34 M H_2_SO_4_ solution and a 0.91 M Li_2_SO_4_ solution (230 mL each) were circulated on the acid and salt sides, respectively. The modified surface of the membrane was positioned facing the acid compartment. The pH of the salt compartment was monitored at regular intervals to determine the change in proton concentration. Based on these measurements, the proton permeability coefficient (*K_p_*) was calculated using Equation (6) [33,34]:

(6) $$K_{p}\left(\frac{m}{s}\right)=\;\frac{k_{v}}{1+k_{V}}\frac{V^{ΙΙ}}{A}\ln\frac{C_{A0}^{Ι}}{C_{A0}^{Ι}-\frac{1+k_{v}}{k_{V}}C_{A}^{ΙΙ}}$$

where $C_{A0}^{Ι}$ and $C_{A}^{ΙΙ}$ are the acid concentrations (mol L^−1^) in the feed (I) and permeate (II) compartments, respectively; *A* is the effective membrane area (m^2^); *t* is the time (s); *V^I^* and *V^II^* are the volumes (mL) of the feed and permeate compartments, respectively; and *k_v_* is the volume ratio of the two compartments (*V^I^*/*V^II^*).

### 2.5. BPED Performance Evaluation

**Figure 3:**
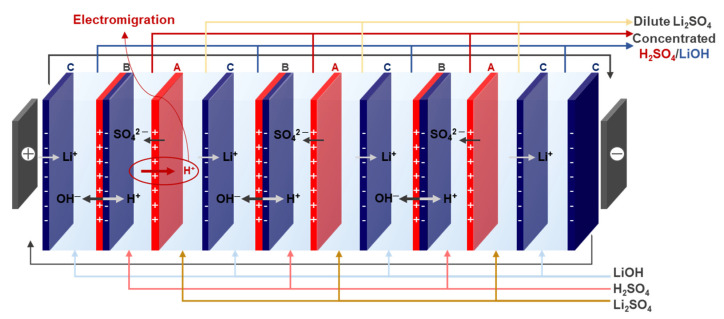
Schematic diagram of the BPED setup and measurement configuration.

The BPED performance was evaluated using a lab-made stack consisting of three cell pairs connected to a power supply (MK6005P, MK Power Corp., Seoul, Republic of Korea). The experiment was conducted under a constant voltage of 10 V. The effective area of the electrodes and membranes was 15 cm^2^, and Pt-plated Ti electrodes were used. Solutions of 1.0 M LiOH (250 mL) were circulated in the electrode chamber, 0.1 M H_2_SO_4_ and 0.1 M LiOH (200 mL each) in the acid and base chambers, and 0.91 M Li_2_SO_4_ (200 mL) in the salt chamber. The flow rate was maintained at 20 mL min^−1^, and the silicone gaskets and PE spacers of a thickness of 1 mm were used. The operating conditions of the BPED system are summarized in Table 1, and the stack configuration is shown in Figure 3. The energy consumption (EC, kWh kg^−1^ LiOH produced) was calculated using Equation (7) [3,35]:

(7) $$EC(\frac{kWh}{{kg}_{LiOH}})=\int_{0}^{t}\frac{UIdt}{{(C}_{t}V_{t}-C_{0}V_{0})M}\;$$

where *U* is the applied voltage (V); *I* is the applied current (A); *V_0_* and *V_t_* are the volumes of the base chamber (L) at 0 and *t* min, respectively; *C_0_* and *C_t_* are the concentration of the base chamber (L) at 0 and *t* min, respectively; and *M* is the molar mass of LiOH (23.94 g mol^−1^). The recovered quantity (*β*, mmol), representing the molar concentration of LiOH produced during the BPED process, was calculated using Equation (8) [5].

(8) $$\beta (mmol)=\frac{C_{t}V_{t}}{M}$$

In addition, the LiOH production rate (PR, kg m^−2^ h^−1^) was calculated using Equation (9) [3,32]:

(9) $$PR(\frac{kg}{m^{2}\cdot h})=\frac{{(C}_{t}V_{t}-C_{0}V_{0})M}{t\;N\;A\times 1000}$$

where *N* is the number of cell pairs, and *A* is the effective area of a single membrane (m^2^).

**Table 1:** Experimental parameters and operating conditions used in the BPED process for LiOH production.

**Electrode**	Pt@Ti plates (area = 15 cm^2^)
**Electrode solution**	1.0 M LiOH (250 mL)
**Feed solution**	0.1 M H_2_SO_4_ (acid, 200 mL) 0.1 M LiOH (base, 200 mL) 0.91 M Li_2_SO_4_ (salt, 200 mL)
**Gasket**	1 mm-thick silicon
**Spacer**	1 mm thick
**Flow rate**	20 mL min^−1^
**Cell pairs**	3 cell pairs

## 3. Results and Discussion

**Figure 4:**
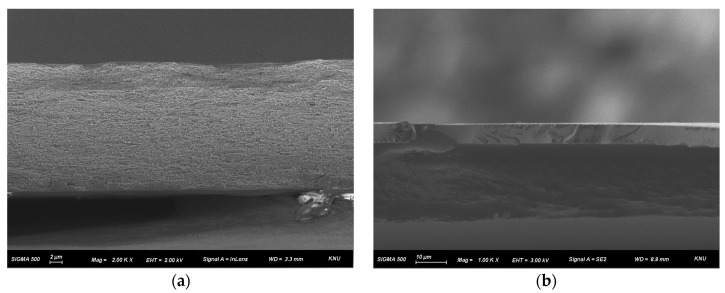
Cross-sectional FE-SEM images of (**a**) pristine PFAEM and (**b**) PPO-diamine-modified composite AEM.

Cross-sectional FE-SEM analysis was conducted to examine the morphological characteristics of the fabricated membranes. As shown in Figure 4a, the porous structure of the PE support film is clearly observed, confirming that its pores were completely filled with the ionomer. In addition, the cross-sectional image of the composite membrane in Figure 4b indicates that the PPO–diamine proton-blocking layer was uniformly formed across the entire membrane surface. The analysis revealed that the base membrane had a thickness of approximately 25 μm, while the proton-blocking layer on top was about 7 μm thick (Figure S2).

**Figure 5:**
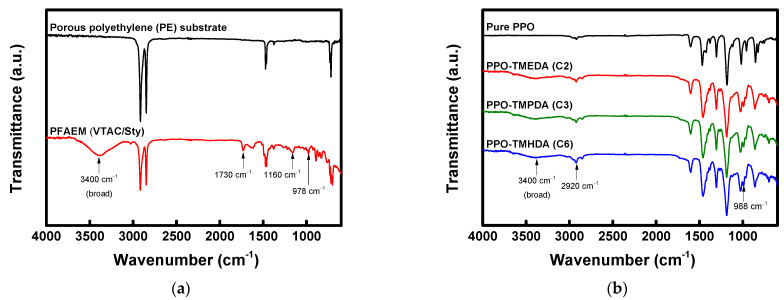
FTIR spectra of (**a**) base membrane (porous substrate and PFAEM: poly(VTAC/Sty)) and (**b**) active layers of PPO-diamine-modified composite AEMs (PPO-TMEDA, PPO-TMPDA, and PPO-TMHDA).

FT-IR spectra were obtained to analyze the chemical structures of the base membrane and the composite membranes, and the results are presented in Figure 5. Figure 5a compares the FT-IR spectra of the porous PE support and the PFAEM. Due to the presence of quaternary ammonium functional groups in the ionomer filled within the PE matrix, the PFAEM spectrum exhibited characteristic absorption peaks at 978 cm^−1^ (C–N), 1160 cm^−1^ (C–O–C), 1730 cm^−1^ (C=O), and 3100–3700 cm^−1^ (O–H) [24,36]. Figure 5b shows the FT-IR spectrum of the PPO–diamine layer, where the characteristic peaks of the PPO backbone and the diamine segments appeared simultaneously. In particular, absorption bands corresponding to C–N (tertiary amine and quaternary ammonium) and C–C (alkyl chain) bonds formed by the combination of PPO and diamine were identified at 988 and 2920 cm^−1^, respectively [37,38,39].

**Figure 6:**
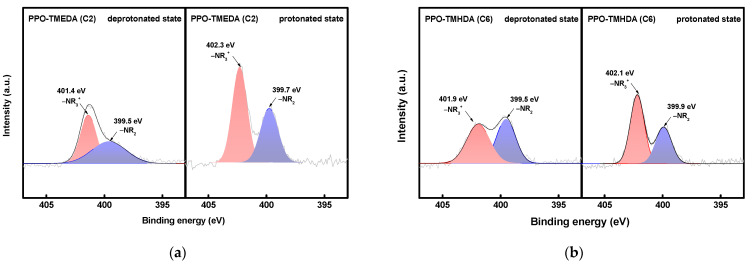
XPS spectra of (**a**) PPO-TMEDA(C2) and (**b**) PPO-TMHDA(C6) in deprotonated and protonated states.

XPS analysis was conducted to investigate the chemical states of PPO–TMEDA and PPO–TMHDA according to the alkyl chain length, and the N 1s spectra are shown in Figure 6. Figure 6a presents the XPS spectra of the deprotonated and protonated states of PPO–TMEDA. The peaks at 399.5 and 399.7 eV correspond to tertiary amine (–NR_2_) groups, while those at 401.4 and 402.3 eV correspond to quaternary ammonium (–NR_3_^+^) groups. Similarly, the spectrum of PPO–TMHDA shown in Figure 6b exhibits –NR_2_ peaks at 399.5 and 399.9 eV, and –NR_3_^+^ peaks at 401.9 and 402.1 eV. These results indicate that the bromine groups in BPPO were substituted with diamines, resulting in a structure containing both tertiary amine and quaternary ammonium groups. Under acidic conditions, the tertiary amine is protonated to form –NR_2_H^+^ species [20,40,41,42]. When protonation occurs, the tertiary amine functional group accepts a proton and is converted to the –NR_2_H^+^ form, which decreases the electron density around the nitrogen atom. As a result, the N 1s peak shifts more noticeably toward higher binding energy. Notably, a larger peak shift was observed for TMEDA than for TMHDA, which can be attributed to the higher degree of protonation [43]. In this study, the degree of protonation (DP) was calculated from the area ratio of the tertiary amine (–NR_2_) peaks in the deprotonated and protonated states, as defined by Equation (10). DP represents the decrease in the –NR_2_ peak intensity in the protonated state relative to that in the deprotonated state, thereby indicating the extent of protonation of tertiary amine groups.

(10) $$DP(\%)=\frac{\Phi_{D,-{NR}_{2}\;}-\Phi_{P,-{NR}_{2}\;}}{\Phi_{D,-{NR}_{2}\;}}\times 100$$

Here, $\Phi_{D,-{NR}_{2}}$ is the –NR_2_ fraction in the deprotonated state, and $\Phi_{P,-{NR}_{2}}$ is the –NR_2_ fraction in the protonated state. The calculation results showed that TMEDA, which has a shorter alkyl chain, exhibited a DP value of 22.8%, whereas TMHDA exhibited a lower DP value of 17.2%. This difference is attributed to the shorter alkyl chains of TMEDA, which decrease the intermolecular distance and free volume, thereby increasing the frequency of proton collisions within the membrane and facilitating more active protonation.

**Figure 7:**
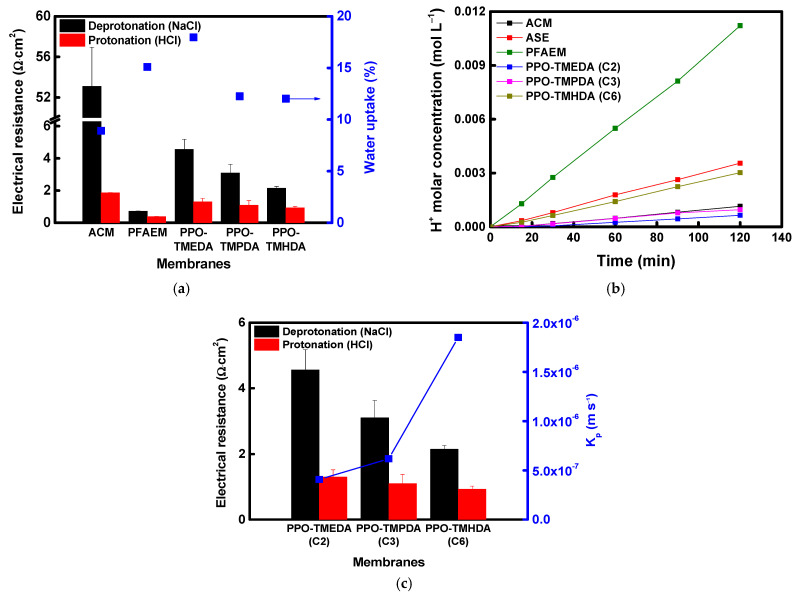
(**a**) Electrical resistance and water uptake of AEMs in deprotonated and protonated states, (**b**) comparison of acid-blocking performance between commercial and prepared AEMs, and (**c**) correlation between electrical resistance and acid-blocking performance of prepared AEMs.

Meanwhile, the physical and electrochemical properties of the fabricated membranes and the commercial ACM were evaluated, and the results are summarized in Table 2. The electrical resistance of the composite membranes increased compared with that of the base membrane due to the introduction of the proton-blocking layer. Nevertheless, the composite membranes exhibited substantially lower electrical resistance than the commercial ACM membrane under identical evaluation conditions. As the alkyl chain length of the diamine increased, the larger free volume tended to lower the resistance. In addition, the longer and more hydrophobic alkyl chains reduced the membrane’s water content [44,45,46]. In this study, the alkyl chain length was limited to C6, based on the findings of a previous study by Hu et al. In their work, conductivity increased as the alkyl chain length of the diamine increased from C4 to C8 but decreased at C10 and beyond. This behavior was attributed to the fact that excessively long chains promote hydrophobic aggregation, which disrupts the continuity of the phase-separated structure and restricts the pathways available for ion transport [47]. When reacted with diamines at the same molar concentration, PPO modified with a lower molecular weight diamine exhibited higher IEC values and a corresponding increase in transport number. As shown in Figure 7a, the membrane resistance under acidic conditions was significantly lower than that under neutral conditions because protonation of the terminal tertiary amine increased the number of positively charged sites. Meanwhile, the proton-blocking performance results (Figure 7b) indicate that surface modification markedly enhanced proton-blocking capability, particularly for membranes with shorter alkyl chain lengths. Among them, the PPO-TMEDA–modified AEM exhibited the highest DP, resulting in strong proton rejection and superior proton-blocking performance compared with the commercial ACM membrane. As shown in Table 2, PPO-TMEDA(C2) has a higher IEC and therefore contains a relatively larger number of ionic functional groups under the same membrane fabrication conditions. This results in a slight increase in WU (approximately 12–18%). However, as discussed above, TMEDA exhibits a higher DP than TMHDA under acidic conditions. This increases the positive charge density within the membrane, strengthens electrostatic repulsion against protons, and ultimately becomes the dominant factor compared to any proton transport enhancement caused by increased WU. Therefore, the improved proton-blocking performance of the PPO-TMEDA(C2) membrane is primarily attributed to the enhanced electrostatic repulsion derived from its higher DP. The correlation between electrical resistance and acid permeability of the fabricated composite AEMs is summarized in Figure 7c. The experimental results clearly demonstrate a trade-off relationship between electrical resistance and acid permeability, which depends on the length of the diamine alkyl chain.

**Figure 8:**
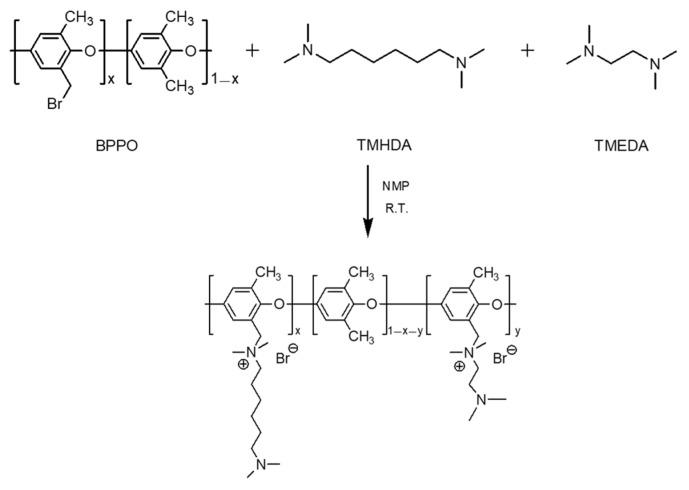
Chemical structure of PPO-TMHDA–TMEDA copolymer.

Since membrane electrical resistance and proton-blocking performance generally exhibit a trade-off relationship, TMHDA (low resistance) and TMEDA (high proton-blocking) were mixed at various molar ratios to achieve balanced performance. Composite membranes were fabricated by adjusting the TMHDA:TMEDA molar ratios to 10:0, 9:1, 7:3, 5:5, and 0:10, and the corresponding structure is illustrated in Figure 8. It should be noted that high TMEDA contents led to gelation during the modification reaction; therefore, experiments were conducted only within the specified composition range.

**Figure 9:**
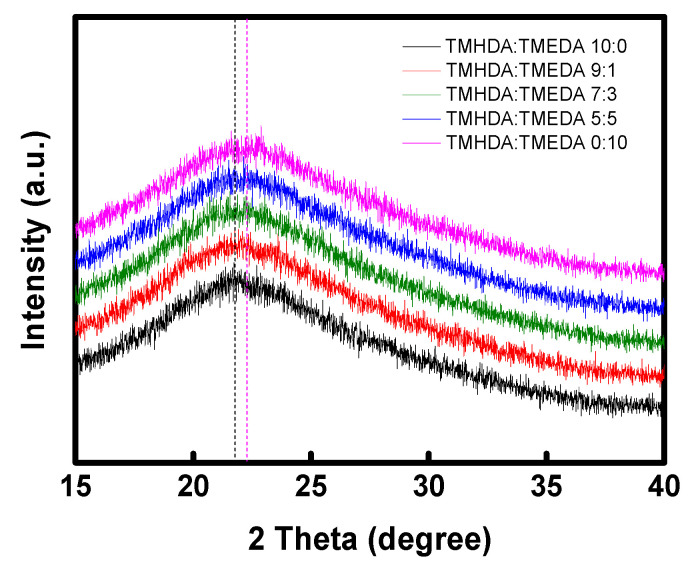
XRD patterns of the prepared PPO-diamine polymers. The guiding lines refer to the peak positions of TMHDA:TMEDA 10:0 (black dashed line) and TMHDA:TMEDA 0:10 (pink dashed line) in the XRD profiles.

To investigate the structural characteristics of the modified PPO–diamines, XRD analysis was conducted, and the results are presented in Figure 9. The interchain distance (*d*-spacing) was calculated using Bragg’s law (Equation (11)) and the values are summarized in Table 3. In general, a smaller *d*-spacing value indicates denser packing of polymer chains around the ionic groups, corresponding to a reduction in the free fractional volume (FFV). As the TMEDA content increased, the *d*-spacing gradually decreased, which can be attributed to the shorter alkyl chain length of TMEDA that results in a shorter distance between polymer chains [44,48,49]. Conversely, as the proportion of TMHDA with bulky alkyl groups increases, interchain interactions decrease, and the polymer chains become more loosely packed, resulting in an increase in FFV. This higher FFV can expand ion transport pathways and enhance membrane flexibility due to the relatively relaxed polymer structure.

(11) nλ=2dsinΘ 

Here, *n* is the order of reflection (–), *λ* is the X-ray wavelength (1.542 Å), *d* is the interplanar spacing or lattice distance (Å), and *Θ* is the diffraction angle (^o^).

**Table 3:** Interchain *d*-spacing values of the PPO-diamine polymers with various diamine compositions.

Samples	TMHDA(C6):TMEDA(C2)				
	10:0	9:1	7:3	5:5	0:10
*d*-spacing (Å)	4.08	4.05	4.03	4.02	3.98

**Figure 10:**
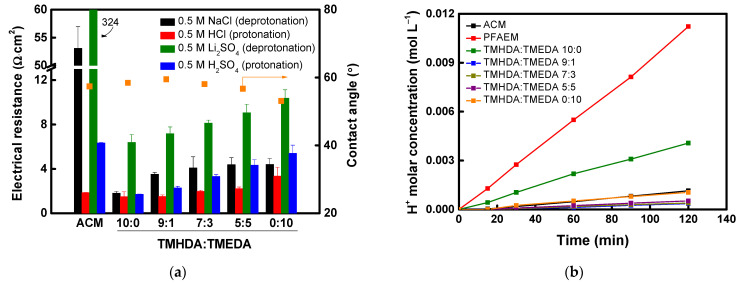
(**a**) Electrical resistance and contact angle of AEMs, and (**b**) comparison of proton-blocking performance between commercial and prepared AEMs.

The ion conductivity of composite membranes incorporating a PPO–diamine-modified layer with varying TMHDA:TMEDA molar ratios was evaluated in different electrolyte solutions. Surface contact angles were also measured to assess changes in hydrophilicity with varying diamine composition. The results are shown in Figure 10a. Both the commercial membranes and the fabricated composite membranes exhibited high resistance under deprotonation conditions and low resistance under protonation conditions. Overall, the fabricated composite AEMs showed significantly lower electrical resistance than the commercial membrane under most conditions. Furthermore, increasing the TMEDA content narrowed the interchain spacing, reducing the FFV, which restricted ion diffusion pathways and increased membrane resistance. Figure 10b displays the proton permeation characteristics of the commercial and fabricated membranes. The results indicate that increasing the TMHDA content enhanced hydrophobicity due to the longer alkyl chains, which improved the proton-blocking performance by inhibiting proton transfer. Among the tested compositions, the TMHDA:TMEDA = 9:1 membrane exhibited the highest proton-blocking capability. This behavior is attributed to the combined effect of increased hydrophobicity from TMHDA and enhanced protonation from TMEDA. Therefore, considering both electrical resistance and proton-blocking performance, the TMHDA:TMEDA = 9:1 composition was determined to be optimal.

Meanwhile, chronopotentiometry experiments were conducted to evaluate the chemical stability of both commercial and prepared AEMs in a strongly acidic solution, and the results are presented in Figure S3. Among various methods for assessing chemical stability, the membrane’s stability was evaluated by performing chronopotentiometry in a highly acidic solution. Because the AEM used in BPED is exposed to strongly acidic environments, its chemical stability under such conditions was considered important. The experimental results showed that the PPO-TMHDA:TMEDA (9:1) composite AEM exhibited a relatively low membrane potential, and both membranes maintained stable potentials without any sudden voltage increase. These results indicate that the prepared AEM exhibits chemical stability in strongly acidic solutions comparable to that of the commercial membrane.

**Figure 11:**
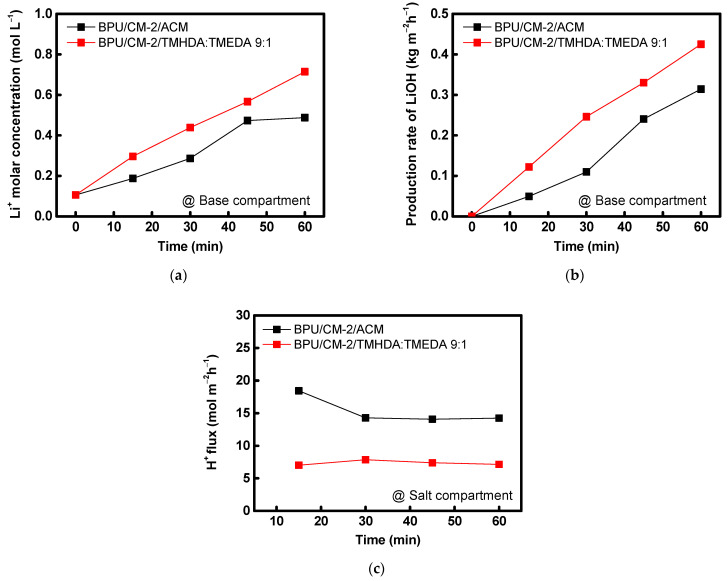
(**a**) Li^+^ concentration in the base compartment, (**b**) LiOH production rate in the base compartment, and (**c**) proton flux measured in the salt compartment during BPED operation using ACM and PPO-TMHDA:TMEDA (9:1)-modified PFAEM.

To verify the performance of the composite AEM fabricated under optimal conditions, it was applied in the BPED process. In this experiment, the BPED performance of the PPO-TMHDA:TMEDA (9:1) composite AEM developed in this study was compared with that of a commercial proton-blocking membrane, ACM. The results are summarized in Figure 11 and Table 4. During BPED operation for LiOH production, protons leaking from the acid chamber into the salt chamber coexist with Li^+^ ions and subsequently migrate to the base chamber through the CEM. Because H^+^ ions are smaller and more mobile, they migrate faster than Li^+^, typically leading to a lower LiOH concentration in the base chamber. However, when the PPO-TMHDA:TMEDA (9:1) composite AEM, which exhibits superior proton-blocking performance compared with the commercial membrane, was employed, proton leakage from the acid chamber to the base chamber was significantly reduced. Figure 11c also presents a graph showing the proton flux changes in the salt chamber during the BPED experiment. The experimental results showed that the proton flux of the membrane developed in this study was half that of the ACM, and both membranes exhibited nearly constant proton flux values after 30 min. Consequently, the selective migration of Li^+^ ions was enhanced, resulting in a higher LiOH concentration. Furthermore, analysis of energy consumption and current efficiency revealed that the PPO-TMHDA:TMEDA (9:1) composite AEM achieved lower energy consumption and faster LiOH production than the commercial membrane. These results demonstrate that the superior proton-blocking performance and high anion conductivity of the PPO–diamine-modified composite AEM significantly enhance the efficiency of the BPED process. Moreover, during the BPED experiments conducted under highly acidic conditions, no peeling or blistering of the coating layer on the prepared AEMs was observed. Therefore, the AEMs fabricated in this study was determined to possess sufficient interfacial stability in highly acidic solutions.

## 4. Conclusions

In this study, a composite AEM suitable for the BPED process for LiOH production was developed using a PFAEM, which consists of a porous PE support impregnated with an anion-exchange polymer, as the base membrane and a PPO-based proton-blocking layer. The proton-blocking layer was fabricated by reacting brominated PPO with diamines of different alkyl chain lengths (TMEDA, TMPDA, and TMHDA), resulting in the coexistence of quaternary ammonium and tertiary amine groups. Increasing the diamine alkyl chain length enlarged the membrane free volume, thereby reducing electrical resistance; however, it also facilitated proton transport, leading to lower proton-blocking performance. Conversely, shorter chains increased membrane resistance but promoted protonation, yielding superior proton-blocking characteristics. To overcome this trade-off, TMHDA (providing low electrical resistance) and TMEDA (providing strong proton-blocking capability) were combined in varying molar ratios to prepare the proton-blocking layer. The optimal composition was obtained at the TMHDA:TMEDA molar ratio of 9:1, which exhibited the lowest membrane resistance and the highest proton-blocking performance. This result is attributed to the synergistic effect of enhanced protonation by the short TMEDA chains and inhibition of proton transport by the hydrophobic long TMHDA chains. Consequently, the PPO–TMHDA:TMEDA (9:1) composite AEM demonstrated significantly lower electrical resistance and superior proton-blocking properties compared with the commercial proton-blocking membrane, ACM. Furthermore, applying the optimized composite AEM to the BPED process resulted in lower energy consumption and enhanced LiOH production efficiency. These results confirm that the proposed PPO–diamine-based composite AEM effectively overcomes the inherent trade-off between proton-blocking performance and ion conductivity, demonstrating its strong potential as a key material for high-efficiency lithium recovery systems.

## Figures and Tables

**Table 2:** Basic properties of the commercial AEM (ACM) and the prepared AEMs.

Membrane	ER (Ω·cm^2^)		*t^−^* (−)	IEC (meq. g^−^^1^)	WU (%)
	0.5 M NaCl	0.5 M HCl			
ACM (Astom)	53.1 ± 3.85	1.87 ± 0.008	0.963	0.61 ± 0.04	8.91 ± 0.06
PFAEM	0.720 ± 0.01	0.39 ± 0.003	0.979	1.80 ± 0.08	16.5 ± 0.93
PPO-TMEDA(C2)	4.56 ± 0.62	1.30 ± 0.22	0.974	2.03 ± 0.14	18.0 ± 1.98
PPO-TMPDA(C3)	3.10 ± 0.53	1.10 ± 0.28	0.969	1.99 ± 0.13	12.2 ± 0.92
PPO-TMHDA(C6)	2.15 ± 0.11	0.93 ± 0.09	0.967	1.92 ± 0.05	12.0 ± 1.10

**Table 4:** Comparison of energy consumption, LiOH production rate, and recovered quantity between ACM and PPO-TMHDA:TMEDA (9:1)-modified PFAEM in BPED.

Membrane	Energy Consumption (kWh kg_LiOH_^−1^)	Production Rate (kg m^−2^h^−1^)	Recovered Quantity (mmol)
ACM (Astom)	7.06	0.314	29.4
TMHDA:TMEDA = 9:1	5.85	0.425	39.7

## Data Availability

The original contributions presented in this study are included in the article/Supplementary Materials. Further inquiries can be directed to the corresponding author.

## Supplementary Materials

The following supporting information can be downloaded at: https://www.mdpi.com/article/10.3390/membranes16010008/s1, Figure S1: ^1^H NMR spectra of (a) PPO and (b) BPPO, Figure S2: Cross-sectional FE-SEM images PPO-diamine-modified composite AEM, Figure S3: Chronopotentiometry curves of commercial and prepared AEMs measured at 30 mA cm^−2^ in 1 M H_2_SO_4_ to evaluate chemical stability.
