# Supplementary material for: Proton-Blocking Anion-Exchange Membranes for Efficient Lithium Hydroxide Recovery by Bipolar Membrane Electrodialysis
Source: Membranes (Basel). 2025 Dec 30;16(1):8. doi: 10.3390/membranes16010008 (PMC12843717; doi:10.3390/membranes16010008)
Supplement: Supplementary file 1 [file membranes-16-00008-s001.zip › membranes-4006764-supplementary.pdf]

**Supplementary materials**

# Proton-blocking anion-exchange membranes for efficient lithium hydroxide recovery by bipolar membrane electrodialysis

Ji-Hyeon Lee and Moon-Sung Kang*

*Department of Green Chemical Engineering, Sangmyung University, Cheonan-si, Chungcheongnam-do 31066, Republic of Korea*

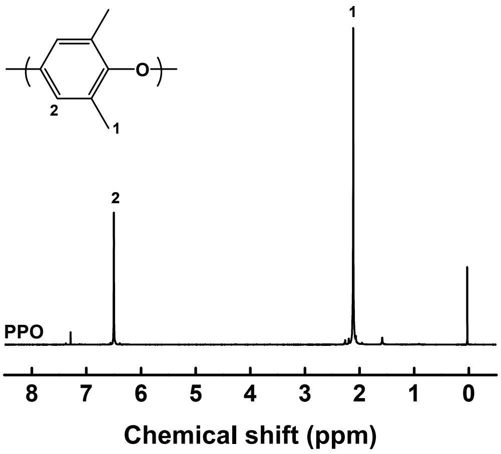

(a)

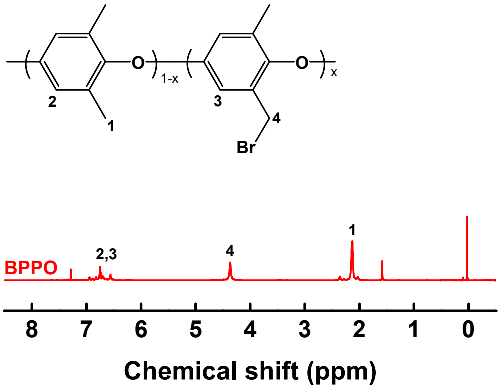

(b)

**Figure S1.** $^1$H NMR spectra of (a) PPO and (b) BPPO.

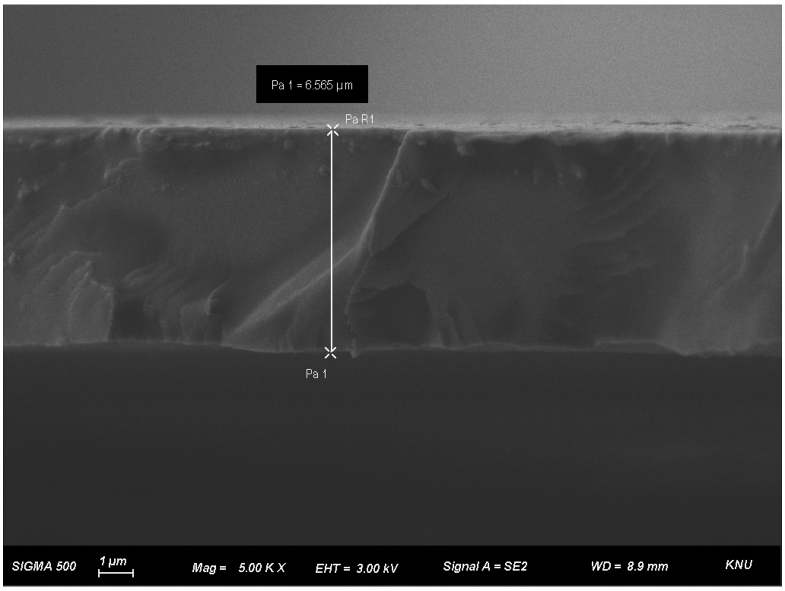


**Figure S2.** Cross-sectional FE-SEM images PPO-diamine-modified composite AEM.

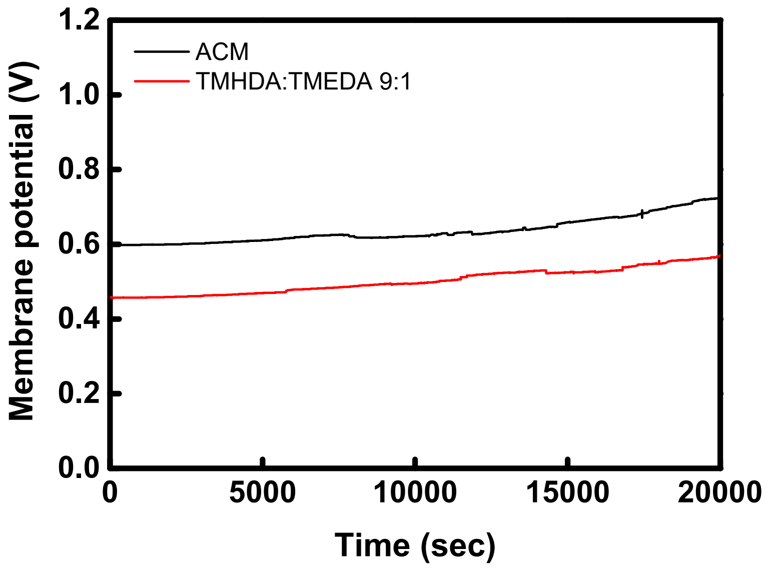


**Figure S3.** Chronopotentiometry curves of commercial and prepared AEMs measured at 30 mA $cm^{-2}$ in 1 M $H_2SO_4$ to evaluate chemical stability.
